# Maternal Smoking, GSTM1 and GSTT1 Polymorphism and Susceptibility to Adverse Pregnancy Outcomes

**DOI:** 10.3390/ijerph6031282

**Published:** 2009-03-26

**Authors:** Regina Grazuleviciene, Asta Danileviciute, Ruta Nadisauskiene, Jone Vencloviene

**Affiliations:** 1 Vytautas Magnus University, K. Donelaicio g. 58, LT-44248, Kaunas, Lithuania; E-Mails: a.danileviciute@gmf.vdu.lt (A.D.); j.vencloviene@gmf.vdu.lt (J.V.); 2 Kaunas University of Medicine, Eiveniu g. 2, LT-50009, Kaunas, Lithuania; E-Mail: Ruta.nadisauskiene@kmuk.lt

**Keywords:** Tobacco smoking, GSTM1, GSTT1 polymorphism, low birth weight risk, fetal growth restriction

## Abstract

The objective of the study was to investigate the association between maternal smoking, GSTM1, GSTT1 polymorphism, low birth weight (LBW, < 2,500 g) and intra-uterine growth restriction (IUGR, < 2,500 g and gestation ≥ 37 weeks) risk. Within a prospective cohort study in Kaunas (Lithuania), a nested case-control study on LBW and IUGR occurrence among 646 women with genotyping of GSTT1 and GSTM1 polymorphisms who delivered live singletons was conducted. Multivariate logistic regression analysis was used to study the association of maternal smoking and polymorphism in two genes metabolizing xenobiotics. Without consideration of genotype, light-smoking (mean 4.8 cigarettes/day) during pregnancy was associated with a small increase in LBW risk, adjusted OR 1.21; 95% CI 0.44 – 3.31. The corresponding odds for IUGR risk was 1.57; 95% CI 0.45 – 5.55. The findings suggested the greater LBW risk among light-smoking mothers with the GSTM1-null genotype (OR 1.91; 95% CI 0.43 – 8.47) compared to those with GSTM1-present genotype (OR 1.11; 95% CI 0.26 – 4.47). When both GSTM1 and GSTT1 genotypes were considered, the synergistic effect was found among smoking mothers: GSTT1-present and GSTM1-null genotype OR for LBW was 3.31; 95% CI 0.60–18.4 and that for IUGR was 2.47; 95% CI 0.31 – 13.1. However there was no statistically significant interaction between maternal smoking, GSTT1- present and GSTM1-null genotypes for LBW (OR 1.45; 95% CI 0.22 – 10.1, p = 0.66) and for IUGR (OR 1.10; 95% CI 0.10 – 12.6, p = 0.93). The results of this study suggested that smoking, even at a low-level, ought to be considered a potential risk factor for adverse birth outcomes and that genetic polymorphism may contribute to individual variation in tobacco smoke response.

## 1. Introduction

Tobacco smoking is known to be associated with adverse pregnancy outcomes. The root causes of many adverse pregnancy outcomes are not well understood, but there is growing evidence that the environment can play an important role. Environmental factors that may have such effects include tobacco smoking, socioeconomic disparities, ambient air pollution, and various other agents encountered both indoors and outdoors [1]. Recent epidemiologic studies have showed, that many adverse pregnancy outcomes might arise from the complex interactions between genes and environment as a function of the age- or stage of development of the individual [2,3].

Active maternal smoking has been associated with a number of adverse reproductive outcomes [4]. Among them are the increased risk of low birth weight (LBW) [5–7], intra-uterine growth restriction (IUGR) [8,9], and, to lesser extent, preterm birth [10]. Numerous studies have found that infants born to smokers weigh substantially less than infants born to nonsmokers [11,12]. Even environmental tobacco smoke (ETS) has been shown to have a negative impact on birth weight. Among women who were exposed to ETS at home and work, infants were lower in weight at delivery in comparison with women who were never exposed to smoke, and even lower in weight when compared with women who smoked during pregnancy [6,7]. Consequently, ETS is recognized as a risk factor for reduction in birth weight and preterm birth of infants [10].

Tobacco smoke is a known to be toxic to humans. It contains over 3,000 chemicals of which over 200 are regarded as poisons and 50 as possible carcinogens [13]. It is generally accepted that there is no safe level of exposure to cigarette smoke [14]. Maternal smoking during pregnancy can result in both pregnancy complications and reduced size of the fetus and neonate. Among women who smoke, genetic susceptibility to tobacco smoke is also a likely causative factor in adverse pregnancy outcomes [15]. Smoking has an even stronger impact on birth weight than alcohol, and today maternal cigarette smoking has been identified as the single largest modifiable risk factor for IUGR in developed countries [16]. However, not all women who smoke cigarettes during pregnancy have LBW infants. The reason for this variability is largely unknown, but may be related to maternal genetic susceptibility [17].

Tobacco smoke is a complex mixture that contains, among other substances, polycyclic aromatic hydrocarbons (PAHs) and *N*-nitrosamines. Recent studies have shown that there are associations between exposure to PAHs and reduced fetal growth and preterm birth [1]. One study in the Czech Republic found that increasing PAH levels during the first month of pregnancy increased the risk of fetal growth restriction [9]. Both PAHs and *N*-nitrosamines are genotoxic and carcinogenic, and their metabolic activation leads to the formation of DNA adducts [15].

Several different classes of enzymes take part in the process of xenobiotic metabolism and carry out conjugation reactions such as the well-known glutathione *S*-transferases (GSTs) [18,19]. The GSTs are a polymorph super-gene family of detoxification enzymes that are involved in the metabolism of numerous toxins and provide critical defense against xenobiotics. GSTT1 encoded enzymes are involved in the metabolism and detoxification of PAHs [20–22]. The GSTT1 enzyme is also important in protecting against genotoxic damage, such as sister chromatid exchanges and the formation of hemoglobin adducts due to the ethylene oxide present in tobacco smoke [17]. GSTM1 enzyme encodes a major detoxification phase enzyme that helps detoxify various xenobiotics. Deficiency in GSTM1 activity is caused by homozygous deletion of GSTM1 and leads to various biological consequences [23]. Both GSTM1 - and GSTT1 enzymes exhibit genetic polymorphism (functional- and nonfunctional phenotypes), that have been shown to be related to birth weight of infants [24]. Several allelic variants of polymorphic GSTs show impaired enzyme activity and increase the risk of fetal development, as well as modify the effects of maternal smoking by increasing or decreasing its risk [25]. One of the maternal genetic polymorphisms of GSTM1 - and GSTT1 expression is through modification of oxidative stress caused by maternal exposure to tobacco smoke [26]. Therefore, the expression of different genotypes may lead to varying susceptibility to the adverse pregnancy effects of cigarette smoke.

In this study, we used a nested case-control design to examine the relationship between maternal smoking, the xenobiotic metabolizing gene GSTM1, GSTT1 polymorphism, and LBW, and fetal growth restriction risk. We hypothesized those women with the GSTM1 - and GSTT1 null genotype who are exposed to cigarette smoke during pregnancy are at elevated risk for adverse pregnancy outcomes.

## 2. Methodology

We conducted a prospective cohort study of pregnant women as a part of the European Commission FP6 HiWATE project [27]. This study, called the HiWATE cohort study, was carried out in the city of Kaunas. For genotype analysis, we used a nested case-control design to study the interactions of maternal smoking with GSTM1, GSTT1, and pregnancy outcomes in 646 women. The information on maternal smoking was obtained by means of a questionnaire.

On their first visit to a general practitioner, all pregnant women living in Kaunas between 2007 and 2008 were invited to join the cohort. We recruited these women for the prospective cohort study, enrolling them at first trimester of gestation at the four prenatal care clinics affiliated to the hospitals of the Kaunas University of Medicine. No compulsion of any kind was imposed on prospective participants for recruitment to the study. Participation was on a voluntary basis and the women were enrolled in the study only if they consented to participate in the cohort. We state that the study ethics comply with the Declaration of Helsinki. The research protocol was approved by the Lithuanian Bioethics Committee and oral informed consent was obtained from all subjects.

Pregnant women of the cohort were asked to answer two questionnaires provided to them at the clinic. The first questionnaire was designed to determine gestational age, maternal-, social, and demographic characteristics, diseases, and health behavior. All participants completed this questionnaire. In all, 3,005 pregnant women were registered of whom 63.9% were eligible and willing to be enrolled into the cohort. Women whose medical records indicated that they had pregnancy-induced hypertension, a history of diabetes mellitus or living outside the Kaunas municipality, were excluded from the study. A special questionnaire was evolved to interview the 1,919 women who agreed to participate; 76.4% of them were interviewed before delivery at hospital and blood samples for genetic analysis was collected. The interviews were conducted by a nurses experienced with this type of work. We also conducted telephone interviews to collect information from those women who agreed to participate in the study but were not interviewed before delivery.

Telephone interviews of about 24% of the total enrolled women were completed within a first month after delivery. Outcomes of interest related to LBW and fetal growth restriction. Pregnancy outcomes were ascertained primary from computerized hospital admission files as well as by abstraction of medical records. Birth weight was abstracted from the birth certificate for all newborns. The age of gestation was calculated using the data of birth as reported on the birth certificate and the 1^st^ day of the last menstrual period as was ascertained at first interview, and by ultrasound examination. We defined newborn weight less than 2,500 g as low birth weight and intra-uterine growth restriction as infants with birth weight less than 2,500 g for those newborns whose gestation period was 37 weeks or longer.

In this study “cases” were defined as women who delivered singleton, live, LBW infants (International Classification of Diseases ten revision (ICD-10), codes P07.0 – 07.1), or IUGR infants (ICD-10, codes P05.0 – 1; P05.9). Controls were defined as women who delivered singleton, live, term infants with birth weight 2,500 g or more.

The genotype analysis group included all women who delivered LBW or IUGR infants and who blood samples for genetic analysis was collected. Random ten controls were identified for every case. Multiple births or newborns with major births defects were excluded.

### 2.1. Exposure Assessment

The interview contained a number of variables including demographics (age, education, family status); reproductive history; job characteristics; self-reported psychosocial stress; health behavior; and diseases. We obtained information about tobacco use in the face-to-face and telephone interviews. We asked the women to report their daily cigarette consumption before pregnancy as well as during pregnancy. We defined “smokers” as those who smoked any number of cigarettes during pregnancy. We compared never smokers with women who smoked during pregnancy.

The self-reported stress of the respondents was assessed by the following thesis: my daily activities are very trying and stressful. Four respondent options were used to define stress: this describes my state (1) very well, (2) fairly well, (3) not very well, (4) not at all. Values 1 and 2 were considered to represent stress; 3 and 4 represented no stress.

The GSTM1- and GSTT1-null genotypes were identified by the multiplex polymerase chain reaction (PCR) in peripheral blood DNA samples. This method allows the detection of the presence of the genotype (at least 1 allele present: AA or Aa) or its absence (complete deletion of both alleles: aa).

Maternal blood samples were collected in vials containing EDTA and stored at a temperature of −20 °C. DNA was purified from the peripheral blood using DNA purification kits (MBI “Fermentas”, Vilnius, Lithuania). DNA concentrations were quantified with a spectrophotometer (Eppendorrf BioPhotometer, 61310488, Hamburg, Germany). A PCR-based study of GSTM1 and GSTT1 polymorphism was carried out according to the method described previously [28]. The primers used for PCR were as follows:

GSTM1 forward 5′-GAA CTC CCT GAA AAG CTA AAG C-3′and reverse 5′-GTT GGG CTC AAA TAT ACG GTG G-3′;
GSTT1 forward 5′-TTC CTT ACT GGT CCT CAC ATC TC-3′ and reverse 5′-TCA CCG GAT CAT GGC CAG CA-3′.

As internal control, a 268-bp fragment of the human β-globin gene was coamplified with a second set of primers (5′-CAA CTT CAT CCA CGT TCA CC-3′) and (5′-GAA GAG CCA AGG ACA GGT AC- 3′) (Biomers.net – the Biopolymer factory, Germany). PCR was carried out in a final volume of 25 μl. The procedure followed for PCR was: primary denaturation at 94 °C for 5 min, denaturation at 94 °C for 1min, annealing at 60 °C for 1 min, extension at 72 °C for 1 min, 30 cycles were conducted. Final extension was at 72 °C for 10 min. The PCR products were electrophoresed in 2% agarose gels and stained in ethidium bromide. The DNA bands were visualised by UV transillumination (EASY Win32, Herolab, Germany). GSTM1 and GSTT1 polymorphisms were coded as present (GSTM1-1 and GSTT1-1) or absent (GSTM1-0 and GSTT1-0).

### 2.2. Statistical Methods

We evaluated tobacco smoke exposure in relation to birth outcomes by calculating crude- and adjusted odds ratios with their 95% confidence intervals (95% CI) for the nested case-control sample. We used logistic regression models to estimate the individual and combined associations of maternal cigarette smoking and GSTM1 and GSTT1 genotypes in relation to newborn LBW and IUGR with adjustment for major covariates.

Comparisons of the associations between smoking and LBW risk factors were made by using Fisher's exact probability test [29]. In logistic regression models for LBW, we assessed a variety of potential confounders, identified from the literature and by univariate analysis. These included: maternal age, pre-pregnancy body mass index (BMI = weight/height^2^) and blood pressure, parity and prior pregnancy history, diseases, education, marital status, employment status and hours worked, stress level, and alcoholic beverage consumption.

Using personal data of the nested case-control sample, we first examined the association between smoking and birth outcomes without consideration of genotypes. Further, we examined the combined association of maternal cigarette smoking and maternal genotypes with birth outcomes controlling for effect of major covariates that changed the adjusted odds ratio for smoking by 10% or more. The subgroups were defined for LBW and IUGR and by maternal smoking status during pregnancy (no vs yes) and genotype for GSTT1 (present vs absent) and GSTM1 (present vs absent). We used chi-square tests to examine the association between genetic polymorphisms and individual susceptibility to tobacco smoking. The gene-cigarette smoke interaction was also tested by adding a product term to the regression models. All the analyses were adjusted for following potential effect modifiers viz. maternal age, BMI, education, and marital status.

## 3. Results and Discussion

### 3.1. Results

Among the pregnant women with smoking and pregnancy outcome data, 71.1% never smoked, 21.5% smoked before but not during pregnancy. Among the women who smoked during pregnancy, light smokers (mean 4.8 cigarettes/day) predominated (92.3% of smokers) and only 7.7% of smokers smoked 10 or more cigarettes per day. In this cohort of women receiving prenatal care at a health maintenance organization, 5.0% of infants had LBW, 5.2% were born preterm, and 2.0% were small for gestational age (intrauterine growth restriction, IUGR).

Table 1 presents maternal characteristics by tobacco smoke-exposure status. This is the overall low-risk population, with the majority of women at their optimal reproductive ages, high education, most having the ideal BMI, blood pressure, and most non-smokers. Smoking during pregnancy was associated with maternal age, education, marital status, and smoking history before pregnancy: the P value of exact test was p < 0.05. Infants of active smokers revealed non-significant reduction in mean birth weight: among non-smokers, the birth weight was 3445 ± 25 g, and light smokers – 3365 ± 59, p = 0.2.

Table 2 presents variables that were associated with maternal smoking and other known LBW risk factors and provides inferential statistics, that is, odds ratios and 95 percent confidence intervals for the discrete variables. In univariate analyses, increasing number of cigarettes smoked was associated with an increased risk in LBW infants. Smokers of 9 cigarettes and more per day had crude odds ratios 1.97 (95% CI 0.78–5.02) times those of unexposed women; however, a small number of LBW cases were reported among smokers and that had an effect on the statistical significance of the results. Age, marital status and blood pressure had statistically significant effect on LBW risk. These risk factors were incorporated into multivariate logistic regression models. Variables that were associated with IUGR risk were same as LBW.

In terms of GSTT1 and GSTM1 genotype frequency, women in the group exposed to tobacco smoke and the groups not exposed were similar. Table 3 presents the combined association of maternal cigarette smoking and maternal genotypes with LBW controlling for effect of major covariates.

The percentage of GSTT1 absent genotype was 16.9% and that of GSTM1 was 46.6%. As shown in Table 3, without consideration of genotype, maternal smoking during pregnancy was associated with an adjusted OR of 1.21 (95% CI 0.44 – 3.31) for LBW compared with the non – smokers. When GSTT1 genotype was considered, the association between maternal smoking and LBW increased and the adjusted OR was 2.06 (95% CI 0.67 – 6.37) among mothers with genotype present, but we could not assess the association among mothers with absent genotype because of 0 LBW cases in the smokers group.

When GSTM1 genotypes were considered, the association between maternal smoking and LBW differed: the adjusted OR was 1.11 (95% CI 0.26 – 4.76) among mothers with present but adjusted OR was 1.91 (95% CI 0.43–8.47) among mothers with absent genotypes. However, a test of interaction between smoking and the GSTM1 – null genotype showed that there was no statistically significant evidence for an effect modification adjusted OR 1.54; 95% CI 0.25 – 9.91, p = 0.59. Presence of both GSTT1 and GSTM1 genotypes tended to increase the smoking effect by 1.49, while the GSTT1 – present genotype and GSTM1 – null genotype were associated with 3.31 times higher risk among smokers (OR 3.31 95% CI 0.60–18.4). A test of interaction between maternal smoking and two studied genotypes did not confer a significant adverse effect on LBW risk, adjusted OR 1.45; 95% CI 0.22 – 10.1, p = 0.66.

Table 4 presents the combined association of maternal smoking and GSTT1 – and GSTM1 genotypes with IUGR. Without considering genotypes, maternal smoking during pregnancy was associated with an adjusted OR of 1.57 (95% CI 0.45–5.55) for IURG compared with the non – smokers.

When we considered genotype GSTT1, the association between tobacco smoke exposure and IURG tended to be higher, and adjusted OR was found to be 2.63 (95% CI 0.65 – 10.6) among the mothers group with GSTT1 genotype present. The estimated smoking effect tendered to be higher among mothers with the GSTM1 – null allele, compared with non-smoking mothers OR was 1.70 (95% CI 0.28 – 10.4). We found some evidence of synergistic effect the GSTT1 and GSTM1 genotypes and active maternal smoking: OR were 2.66 (95% CI 0.38 – 18.5) for both alleles present and OR 2.47 (95% CI 0.31 – 13.1) for GSTM1 absent; nevertheless, there was no statistically significant interaction, adjusted OR 1.10; 95% CI 0.10 – 12.61, p = 0.93.

### 3.2. Discussion

In this molecular epidemiological study on maternal cigarette smoking and genetic determinants of xenobiotic metabolism, we found some evidence that effects of maternal smoking on LBW risk and infant growth were increased by maternal GSTM1 null genotype. This study used a case-control design to analyze the genetic effects and the gene-environment interaction controlling for major confounding variables. Consistent with previous studies, we found that maternal cigarette smoking was associated with fetal growth restriction and increased risk of LBW risk [5,17]. Our findings are consistent with a number of other studies that LBW risk may vary in relation to maternal age, BMI, parity, and other variables of the population in the study [29–31]. Some other investigators who have examined the issue, revealed dose-response gradients in relation to the amount smoked [4,33].

Present our findings show the greater LBW risk among light-smoking mothers with GSTM1 null genotype compared to those with GSTM1 present genotype, however the findings do not show a statistically significant results. These results are consistent with previous studies which analysed genetic susceptibility to cigarette smoke in the context of LBW or IUGR risk.

Wang *et al.* reported that pregnant women with certain genotypes are susceptible to the adverse pregnancy effects of tobacco smoking, such as an increased risk of LBW [17]. Without consideration of genotype, maternal smoking during pregnancy was associated with reduction in birth weight and elevated risk of LBW. When GSTT1 genotype was considered, the reduction in birth weight increased and 1.7 (0.9 – 3.2) - fold elevated risk of LBW for those with the genotype present, and 3.5 (1.5 – 8.3) - fold elevated risk of LBW for GSTT1 genotype absent was found among smoking mothers. The corresponding features for IUGR were 3.3 (1.7 – 6.3) and 2.5 (0.9 – 6.4), suggesting an interaction between metabolic genes and maternal smoking.

It has been reported that an individual difference in metabolic activation and detoxification xenobiotics partly depends on the genetic polymorphisms associated with GSTT1 and GSTM1 enzymes [33]. The interactive effect of exposure to tobacco smoke and the presence of the GSTT1 polymorphism on infant birth weight was found to be significant by multivariate analysis, whereas the interactive effect of the presence the GSTM1 polymorphism did not reach statistical significance (p = 0.21) [25].

Sasaki *et al.* also reported combined effects between maternal genetic polymorphisms and smoking during pregnancy [35]. The effects on reduction birth weight were not observed among women with GSTM1 null genotype who had never smoked. The authors conclude that maternal smoking in combination with maternal genetic susceptibility may adversely affect infant birth weight. However, results presented here do not show a statistically significant association between infant birth size and maternal smoking as linked to the GSTT1 genotype, while birth weight and length were significantly lower in subjects with GSTM1 null genotype.

Sram *et al.* found that the risk of LBW and prematurity was significantly increased by the genotypes of GSTM1 null and a genotype combination with the CYP1A1*2A genotype [36]. A survey among pregnant women have shoved that a combination of the GSTM1 null and the GSTT1 null genotypes exacerbate the effect of maternal exposure to tobacco smoke on birth weight more than the presence of either genotype one [24].

Different results were presented by some authors [25]. In the case-control study, controlling for several confounding factors, the authors revealed that the maternal GSTT1 null genotype had a 1.6 – fold reduced risk for small-for-gestational-age births. However, after adjustment for maternal smoking (categories less than 10 cigarettes/day and more than 10 cigarettes/day) the results were not statistically significant.

There is evidence that effect of cigarette smoke exposure depends on population characteristics: among Japanese GSTM1 null genotype decreases fetal growth but this effect is not observed in Caucasians. Moreover, the adverse effect on birth weight did not always accompany fetal growth restriction [37].

Previous studies have suggested several plausible gene-smoking interaction explanations. First, tobacco smoke could disturb fetal and placental cellular regulation via elevated PAH-DNA adducts due to the increased activity of enzymes that metabolize cigarette toxins (e.g. CYP1A1) and lower or absent activity of enzymes that detoxify these compounds (e.g. GSTT1 and GSTM1 null genotypes) [15]. Second, gene-smoking interactions may exert their synergistic effects through oxidative stress that occurs upon tobacco smoke exposure. In response to this stress various inflammatory cytokines are produced in lung tissue increasing inflammatory responses and immune responses [38]. Moreover, as reported by some authors, maternal exposure to tobacco smoke affects the fetal urine cotinine concentration and also induces production of oxidative stress [26]. Further, other environmental factors and genetic polymorphism of GSTM1 and GSTT1 may modify the response to oxidative stress and lead to adverse pregnancy outcomes [32].

In this study, we demonstrated that there is increase in LBW and IUGR risk among smoking women even after adjusting for maternal age, education, BMI, and marital status; however, these findings suggest that there was no significant association between the GSTT1 and GSTTM1 polymorphism with low-level maternal smoking during pregnancy. The reason may be that the size of our nested case-control study and the proportion of women who smoked during pregnancy were too small to detect any significant difference.

Consistent to previous studies, we found that the effect of tobacco smoke increased LBW risk in the women’s group with combination of GSTT1 present and GSTM1 absent alleles was more than 3 times greater compared with the non-smokers group (OR 3.31; 95% CI 0.6 – 18.4). Similar evidence of the synergic effect of GSTT1 and GSTM1 polymorphism we revealed on fetal growth restriction, adjusted OR 2.47; 95% CI 0.31 – 13.1. The adverse effects of GSTM1 null genotype on IUGR in the presence of cigarette smoke exposure were observed even among light smokers. These data strengthen the previous research findings that indicated that subjects with GSTM1 null genotype have a greater risk of toxic tobacco smoke effects while restricted fetal growth among light smokers provides evidence of unhealthy development in uterus [35].

When the results of this study are interpreted, a few conditions should be taken into account. This is a low-risk population with low-level tobacco smoke exposure, and low prevalence of GSTT1 null genotypes and these factors may limit extrapolation of these results to the other populations. The evaluation of exposure to tobacco smoke was indirect; we used self-reported information on smoking during- and before pregnancy, and thus the possibility of reporting bias exists. Because of the subjective measure of smoking exposure, there is a possibility of random exposure classification errors. However, in this study, we controlled for the main variables that might confound the association between smoking, genetic polymorphism, and birth outcomes, among them age, BMI, education, and family status, therefore, the residual confounding of results by smoking is expected to be small.

Our findings stress the need for appropriate policy and programs aimed at cessation of tobacco use among pregnant women. The evidence of increased risk of adverse birth outcomes in presence of genetic polymorphism reinforces the motivation argument for quitting smoking. This could help in directing smoking cessation interventions toward pregnant women and prevent adverse birth outcomes since smoking prevalence rate and effectiveness of tobacco control programs mostly depend not only on legislative recourses, but also on the individual perceiving that smoking is harmful to health [39,40].

## 4. Conclusions

In summary, we have demonstrated that tobacco smoke exposure, even at a low-level, is associated with fetal growth restriction. Such as association, however, is modified by an individual’s genotype. This study supports the importance of considering genetic susceptibility in prevention of adverse birth outcomes and evaluation of the effectiveness of anti-smoking preventive programs.

## Figures and Tables

**Table 1.** Percent distribution of subjects by smoking for various characteristic and pregnancy outcomes.

Maternal characteristics	Total	Smoking during pregnancy (%)		Exact test

Variables	N	None	Yes	p
Age:				
≤ 20 y	28	71.4	28.6	
21 – 30 y	402	86.8	13.2	
>30 y	216	92.1	7.9	0.004
Education:				
university	309	96.8	3.2	
college and ≤ 12 y	337	79.8	20.2	< 0.001
Marital status:				
married	493	92.3	7.7	
not married	153	73.9	26.1	< 0.001
Parity:				
1^st^	320	89.1	10.9	
2^rd^ and more	326	86.8	13.2	0.38
Pregnancy history:				
no prior	517	87.2	12.8	
losses	129	90.7	9.3	0.28
Gestational age:				
≥ 37 weeks	600	87.5	12.5	
< 37 weeks	46	93.5	6.5	0.23
Blood pressure:				
≤ 140 – 90 mm/Hg	558	87.8	12.2	
> 140/90 mm/Hg	88	88.6	11.4	0.83
Stress:				
no	523	88.5	11.5	
yes	123	85.4	14.6	0.33
Mother diseases:				
no	474	88.8	11.2	0.25
yes	172	85.5	14.5	
Body mass index (BMI):				
normal - overweight (25.1 – 30)	558	87.8	12.2	0.83
obesity (> 30)	88	88.6	11.4	
Smoking before pregnancy:				
none	461	100.0	0.0	
1 – 9 cigs./d.	169	60.4	39.6	
> 9 cigs./d.	16	31.3	68.8	< 0.001
Smoking duration before pregnancy:				
non smoker	461	100.0	0.0	
1 – 5 y	122	66.4	33.6	
6 – 10 y	47	44.7	55.3	
> 10 y	16	31.3	68.7	< 0.001
Mean birth weight (g), ± SD	3,436 ± 24	3,445 ± 25	3,365 ± 59	0.21

**Table 2.** Distribution of maternal characteristics among low birth weight (LBW) cases and controls, odds ratios (OR) and their 95% confidence intervals (CI).

Maternal characteristics	Cases LBW		Controls		Inferential statistics	
	N	%	N	%	OR	95% CI
Age:						
21 – 30 y	26	45.6	376	63.8	1	
≤ 20 y	5	8.8	23	3.9	3.14	1.11 – 8.94
> 30 y	26	45.6	190	32.3	1.98	1.12 – 3.50
Education:						
university	28	49.1	281	47.7	1	
college & < 12 y	29	50.9	308	52.3	0.95	0.55 – 1.63
Marital status:						
married	37	64.9	456	77.4	1	
not married	20	36.1	133	22.6	1.85	1.04 – 3.30
Parity:						
1^st^	26	45.6	294	49.9	1	
2^nd^ and more	31	54.4	295	50.1	1.19	0.69 – 2.05
Previous pregnancy history:						
no prior	42	73.7	475	80.6	1	
losses	15	26.3	114	19.4	1.49	0.80 – 2.78
Blood pressure:						
≤ 120/80 – 140 – 90 mm/Hg	52	91.2	506	85.9	1	
> 140/90 mm/Hg	5	8.8	83	14.1	0.59	0.23 – 1.51
Stress:						
no	45	78.9	478	81.2	1	
yes	12	21.1	111	18.8	1.15	0.59 – 2.24
Mother diseases:						
no	41	71.9	433	73.5	1	
yes	16	28.1	156	16.5	1.08	0.59 – 1.99
Body mass index (BMI):						
BMI > 30	7	12.3	135	22.9	1	
BMI ≤ 30	50	87.7	454	77.1	2.12	0.94 – 4.79
Smoking during pregnancy:						
non smoker	39	68.4	422	71.6	1	
≤ 9 cig.	17	29.8	152	25.8	0.87	0.45 – 1.68
> 9 cig.	1	1.8	15	2.5	1.97	0.78 – 5.02

**Table 3.** Crude and adjusted associations as odds ratios (OR) maternal smoking during pregnancy with low birth weight by maternal genotypes.

Genotype	Smoking status	N	LBW, %	Crude OR 95% CI	Adjusted* OR 95% CI
Total sample	Never	342	8.8		
	Quitter	86	10.5	1.22 0.55 – 2.67	1.18 0.53 – 2.62
	Smoking	52	11.5	1.36 0.54 – 3.44	1.21 0.44 – 3.31

GSTT1	Never	289	9.0		
Present	Smoking	38	15.8	1.90 0.73 – 4.96	2.06 0.67 – 6.37
GSTT1	Never	53	7.5		
Absent	Smoking	14	0		

GSTM1	Never	168	8.9		
Present	Smoking	31	9.7	1.09 0.30 – 4.0	1.11 0.26 – 4.76
GSTM1	Never	174	8.6		
Absent	Smoking	21	14.3	1.77 0.47 – 6.69	1.91 0.43 – 8.47

**Interaction: smoking x GSTM1 absent		OR 1.62 (0.25 – 10.4), p = 0.60; OR* 1.54 (0.25 – 9.91), p = 0.59			

GSTT1 & GSTM1	Never	145	9.7		
Present	Smoking	22	13.6	1.48 0.39 – 5.62	1.49 0.33 – 6.79
GSTT1 present &	Never	144	8.3		
GSTM1 absent	Smoking	16	18.8	2.54 0.63 – 10.2	3.31 0.60 – 18.4

**Interaction: smoking x GSTT1 present & GSTM1 absent		OR 1.72 (0.25 – 11.8), p = 0.58; OR* 1.45 (0.22 – 10.1), p = 0.66			

* Logistic regression model: women BMI ≤ 30, age ≥ 20 years, adjustment for maternal education and marital status.

** Test of interaction: a P value is presented for testing the null hypothesis, odds ratio = 1.0 in logistic regression models for the product term, smoking x genotypes.

**Table 4.** Crude and adjusted associations as odds ratios (OR) maternal smoking during pregnancy with intrauterine fetal growth restriction by maternal genotypes.

Genotype	Smoking status	N	LBW, %	Crude OR 95% CI	Adjusted* OR 95% CI
Total sample	Never	325	4.0		
	Quitter	80	3.8	0.94 0.26 – 2.36	0.85 0.23 – 3.10
	Smoking	50	8.0	2.09 0.65 – 6.68	1.57 0.45 – 5.55

GSTT1	Never	274	4.0		
Present	Smoking	36	11.1	2.99 0.90 – 9.94	2.63 0.65 – 10.6
GSTT1	Never	51	3.9		
Absent	Smoking	14	0		

GSTM1	Never	158	3.2		
Present	Smoking	30	6.7	2.19 0.40 – 11.8	2.00 0.30 – 13.2
GSTM1	Never	167	4.8		
Absent	Smoking	20	10.0	2.21 0.44 – 11.2	1.70 0.28 – 10.4

**Interaction: smoking x GSTM1 absent		OR 1.01 (0.10 – 10.5), p = 0.99; OR* 0.98 (0.09 – 10.3), p = 0.99			

GSTT1 & GSTM1	Never	136	3.7		
Present	Smoking	21	9.5	2.76 0.50 – 15.2	2.66 0.38 – 18.5
GSTT1 present &	Never	138	4.3		
GSTM1 absent	Smoking	15	13.3	3.39 0.62 – 18.5	2.47 0.31 – 13.1

**Interaction: smoking x GSTT1 present & GSTM1 absent		OR 1.23 (0.11 – 13.7), p = 0.87; OR* 1.10 (0.10 – 12.6), p = 0.93			

* Logistic regression model: women BMI ≤ 30, age ≥ 20 years, adjustment for maternal education and marital status.

** Test of interaction: a P value is presented for testing the null hypothesis, odds ratio =1.0 in logistic regression models for the product term, smoking x genotypes.
